# Lesion Activity on Brain MRI in a Chinese Population with Unilateral Optic Neuritis

**DOI:** 10.1371/journal.pone.0141005

**Published:** 2015-10-20

**Authors:** Chuntao Lai, Qinglin Chang, Guohong Tian, Jiawei Wang, Hongxia Yin, Wu Liu

**Affiliations:** 1 Department of Neurology, Beijing Tongren Hospital, Capital Medical University, Beijing, China; 2 Department of Radiology, Beijing Tongren Hospital, Capital Medical University, Beijing, China; 3 Ophthalmology Center, Beijing Tongren Hospital, Capital Medical University, Beijing, China; Instituto Cajal-CSIC, SPAIN

## Abstract

Longitudinal studies have shown that brain white matter lesions are strong predictors of the conversion of unilateral optic neuritis to multiple sclerosis (MS) in Caucasian populations. Consequently brain MRI criteria have been developed to improve the prediction of the development of clinically definite multiple sclerosis (CDMS). In Asian populations, optic neuritis may be the first sign of classical or optic-spinal MS. These signs add to the uncertainty regarding brain MRI changes with respect to the course of unilateral optic neuritis. The aim of this study was to examine the association between brain lesion activity and conversion to CDMS in Chinese patients with unilateral optic neuritis. A small prospective cohort study of 40 consecutive Chinese patients who presented with unilateral optic neuritis was conducted. Brain lesion activity was recorded as the incidence of Gd-enhanced lesions and new T2 lesions. Brain lesions on MRI that were characteristic of MS were defined according to the 2010 revisions of the McDonald criteria. The primary endpoint was the development of CDMS. We found that nineteen patients (48%) had brain lesions that were characteristic of MS on the initial scan. One of these patients (3%) had Gd-enhanced brain lesions. A significantly lower percentage of the patients (10%, *p*<0.001) presented with new T2 brain lesions on the second scan. During a median of 5 years of follow-up, seven patients (18%) developed CDMS. There was no significant difference in the conversion rate to CDMS between patients with and without brain lesions that were characteristic of MS (4/19 and 3/21, respectively; Fisher exact test, one-sided, *p* = 0.44). We conclude that brain lesions characteristic of MS are common in Chinese patients with unilateral optic neuritis; however, these patients exhibit low lesion activity. The predictive value of brain lesion activity for CDMS requires investigation in additional patients.

## Introduction

Longitudinal studies have shown that brain white matter lesions are strong predictors of the conversion of unilateral optic neuritis to multiple sclerosis (MS) in Caucasian populations. The cumulative probability of developing clinically definite multiple sclerosis (CDMS) within 5 or 15 years after the onset of optic neuritis is 51% and 72%, respectively, among those displaying white matter lesions on brain MRI at baseline [1, 2]. Furthermore, a higher rate of conversion to CDMS (89% of 42 patients) was reported after 10 years of follow-up [3]. A recent study found that the presence of lesions characteristic of MS displayed high specificity (>90%) for predicting conversion to CDMS in patients who have experienced a clinically isolated syndrome [4]. That study included 67 patients with unilateral optic neuritis.

Brain MRI criteria have been developed to improve predictions for CDMS in patients who have experienced a clinically isolated syndrome, including those with unilateral optic neuritis. These criteria have focused on the locations, number, and activity of the lesions [5]. Three regions are defined as characteristic for demyelination: juxtacortical, infratentorial, and periventricular. The more recent criteria [6] state that dissemination of the lesion in space (DIS) requires at least one lesion in at least two of four locations (juxtacortical, infratentorial, periventricular and spinal cord). Moreover, the presence of active lesions (Gd-enhanced lesions or new T2 lesions) was strongly associated with the development of CDMS and was added to the MRI criteria for MS. Fewer brain lesions at each location are now required, although the specificity requirement has been maintained. These criteria appear to be similar to the Asian criteria [7]. However, the latter are based on few retrospective studies.

Optic neuritis, which is highly associated with MS in Western patients, usually presents with typical clinical features, including acute, monocular vision loss. However, in Asian populations, optic neuritis may be the first sign of classical or optic-spinal MS. These signs add to uncertainty regarding brain MRI changes with respect to the course of unilateral optic neuritis. The purpose of this study was to examine the association between brain lesion activity on MRI and conversion to CDMS in Chinese patients with unilateral optic neuritis. Because severe vision loss was common in this Asian population, we also measured the anti-aquaporin-4 antibody levels and determined mtDNA mutations to exclude neuromyelitis optica and Leber hereditary optic neuropathy, respectively.

## Methods

### Clinical methods and patients

Forty consecutive patients with unilateral optic neuritis were enrolled in this study. They were observed at the Department of Neurology, Beijing Tongren Hospital, Capital Medical University, China, from July 2006 to June 2008. All patients underwent a neuro-ophthalmological examination by both a neurologist and an ophthalmologist within one month of symptom onset and received a diagnosis of unilateral optic neuritis. Our protocol was approved by the IRB of the Beijing Tongren Hospital, Capital Medical University, and the patients provided written informed consent.

### Diagnosis and classifications

**Fig 1:**
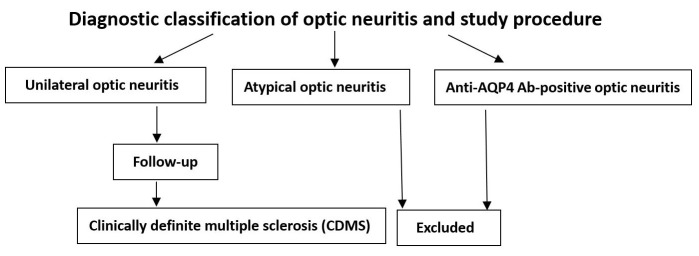
Diagnostic classification of optic neuritis and study procedure. Patients with atypical optic neuritis or who were anti-aquaporin-4 (AQP4) antibody (Ab)-positive were excluded.

Unilateral optic neuritis (**Fig 1**) manifested with unilateral onset, acute or sub-acute loss of vision, pain upon eye movement, abnormal color perception, and a relative afferent pupillary defect (RAPD).

Atypical patients had at least one of four features: simultaneous bilateral onset, vision loss progressing for >2 weeks since onset, no visual recovery within 3 weeks after onset, or worsening of vision by at least one line of acuity after withdrawal of corticosteroids [8, 9].

We ruled out anti-AQP4 Ab-positive, ischemic and compressive patients and those with hereditary and systemic disease-related optic neuropathy based on patient history and auxiliary testing.

Patients with other previous neurological events or receiving disease-modifying therapy were excluded.

We defined CDMS according to the Poser criteria [10] and the 2010 revisions of the McDonald criteria [11]:

1. A new attack, which involved different regions of the central nervous symptom from the optic nerve, was separated by a minimum of one month after optic neuritis, lasted at least 24 hours and was confirmed by examination;
2. A second episode of optic neuritis and DIS on brain MRI.

Considering the specificity and predictive value of the presence of a brain lesion for MS development, we defined brain lesions on MRI that were characteristic of MS according to the 2010 revisions of the McDonald criteria as follows: juxtacortical, periventricular, and infratentorial lesions [11]. Additionally, for each brain lesion that was characteristic of MS, the DIS and the dissemination of the lesion over time (DIT) were defined.

### Auxiliary examination

All patients underwent routine examinations within one month after onset. This examination included an assessment of visual acuity, a Humphrey visual field analysis, a fundus examination, and tests of flash and pattern-reversal visual-evoked potentials. All patients underwent a chest x-ray and brain and optic nerve MRI. Serological testing included measurements of ESR, CRP, RF, syphilitic serology (VDRL and FTA-ABS), ANA and ENA, ANCA, anti-cardiolipin antibodies (ACA), ACE, anti-thyroid antibodies (ATA), mtDNA (11778, 14484 and 3460), and anti-AQP4 Ab. Not all of the patients underwent all of the tests. We tested for anti-AQP4 Abs using an indirect immunofluorescence method and human AQP4-transfected cells.

### MRI scans of the brain and the optic nerve

MRI scans were performed on a GE Twinspeed 1.5 T scanner. The sequences included 6-mm thickness axial T1WI (TR = 1980-2280ms, TE = 23-27ms, Matrix = 384×224), T2WI (TR = 3700-4800ms, TE = 99-106ms, Matrix = 384×224), 5-mm thickness sagittal T2 FLAIR (TR = 8267-8269ms, TE = 127-128ms, Matrix = 320×192), 3-mm thickness coronal T2 STIR (TR = 6000-6200ms, TE = 45ms, Matrix = 320×192), contrast-enhanced imaging via gadolinium (Gd) infusion (0.1 mmol/kg), and 8HRBRAIN. Both an experienced radiologist and a neurologist reviewed all MRI archives using a DICOM viewer program (eFilm Workstation™ 1.8.3). The lesion numbers, locations and sizes were recorded. A third reader (either a radiologist or a neurologist) made the final decision if the first two readers did not reach a consensus. We considered high white matter signals on the axial T2WI and sagittal T2FLAIR sequences to represent demyelinating lesions. The lesion size was not limited.

### Brain lesion activity

The number and location of the active lesions (Gd-enhanced lesions and new T2 lesions on MRI at follow-up) were recorded. The brain lesion activity was recorded as the incidence of Gd-enhanced lesions and new T2 lesions.

### Follow-up

Follow-up brain MRI was performed 3 months after the initial scan.

The patients were followed up for a median of 5 years (range 4–7 years, once per year) via either an outpatient consultation or a phone interview. For patients receiving phone interviews, a second remote clinical event was required to be confirmed locally by a neurologist for diagnosis of the patient with CDMS. The primary endpoint was the development of CDMS.

New attacks that involved the central nervous symptom, including the optic nerve, and events associated with systemic diseases were recorded.

### Statistics

SPSS version 13.0 (SPSS, Chicago, IL, USA) was used to perform the statistical analysis, and Chi-square tests were used in association with Pearson or Fisher exact tests for qualitative data. These tests included comparisons of brain lesion parameters across two separate scans and assessment of the association between the presence of brain lesions characteristic of MS and clinical outcomes. We considered *p* < 0.05 to be statistically significant.

## Results

### Demographic characteristics

The female to male ratio (**Table 1**) was 2.3:1 among the 40 Chinese patients. The demographic differences based on sex were not significant. Syphilitic serology was performed on all patients, and all patients were negative for syphilis. Fifteen patients, including all of the males, underwent an assessment of mtDNA mutations, and all patients were negative for mtDNA mutations. None of the patients had a maternal family history of MS.

**Table 1:** Patient demographic characteristics.

Characteristic	Patients
Sex (n, %)	
Female	28 (70)
Male	12 (30)
Age (years, mean±SD)	28.0±13.0
SON (n, %)	25 (63)
Eye pain (n, %)	28 (70)
Swollen optic disc (n, %)	15 (38)

n, number; SON, severe optic neuritis (visual acuity ≤10/100).

### Initial brain MRI

Twenty-two (55%) patients demonstrated brain lesions (**Table 2**). Nineteen patients (48%) had lesions that were characteristic of MS **(Table 2, S1 Fig)**. Juxtacortical lesions (35%) were the most common location. Seventeen patients with lesions that were characteristic of MS had at least one lesion ≥ 3 mm. The number of lesions that were characteristic of MS ranged from 1 to 21 (mean = 4) per patient. Most of the non-characteristic lesions were located in deep white matter and lobes.

**Table 2:** Brain MRI changes and CDMS conversion.

	UON (n = 40)
MRI abnormalities (n, %)	22 (55)
Lesions characteristic of MS (n, %)	19 (48)
Juxtacortical lesions	14 (35)
Periventricular lesions	8 (20)
Infratentorial lesions	8 (20)
Lesion number	
< 3 mm	27
3 mm-6 mm	48
> 6 mm	8
Gd-enhanced lesions (n, %)	1 (3)
New T2 lesions (n, %)	4 (10)
DIS (n, %)	8 (20)
DIT (n, %)	2 (5)
Non-characteristic lesions (n, %)	17 (43)
CDMS conversion (n, %)	7 (18)
Relapsing optic neuritis	3
Optic-spinal syndrome	4

UON, unilateral optic neuritis; n, number; DIS, dissemination of the lesion in space; DIT, dissemination of the lesion over time.

### Brain lesion activity

All patients with brain lesions were infused with Gd at the initial scan. One (3%) patient showed slight enhancement in a periventricular lesion.

Four patients (10%) showed new T2 lesions (all > 3 mm) in addition to the lesions observed on the first scan. This percentage was significantly lower than the percentage of patients displaying T2 lesions on the first scan (48%; Pearson’s chi-square test, two-sided, *p*<0.001). The first patient, who lacked brain lesions on the first scan, demonstrated a new periventricular lesion on the second scan. The second patient, who met the DIS criteria, showed a new juxtacortical lesion and new periventricular lesions on the second scan. The third patient, who had a juxtacortical lesion on the first scan, developed a new brainstem lesion on the second scan. The fourth patient, who presented with juxtacortical lesions on the first scan, had two new juxtacortical lesions on the second scan.

### Brain lesions characteristic of MS and conversion to CDMS

During a median of 5 years of follow-up, seven patients (18%) converted to CDMS. There was no significant difference in the conversion rate to CDMS between the patients with and without brain lesions that were characteristic of MS (4/19 and 3/21, respectively; Fisher exact test, one-sided, *p* = 0.44). Three patients with relapses of optic neuritis and one patient with partial myelitis showed brain lesions that fulfilled the DIS criteria. Among the 4 patients who presented with optic-spinal syndrome (**Table 3)**, spinal MRI showed that three of these patients had oval or patchy lesions and that one patient had a longitudinal lesion that extended across fewer than 3 spinal cord segments (**S2 Fig**). No patient developed new symptoms or signs associated with systemic diseases during follow-up.

**Table 3:** Clinical, brain MRI and serological characteristics of the 4 optic-spinal syndrome patients.

Case	Onset age (yr)	Vision loss	Partial myelitis	No. of ON	Diplopia	AQP4 Ab	Initial MRI	Second MRI	CDMS conversion (yr)	Latest EDSS score
1	36	severe	Yes	1	No	–	DIS	–	3	1
2	21	severe	Yes	2	No	–	–	–	3	2
3	24	severe	Yes	1	Yes	–	–	–	1	0
4	29	severe	Yes	3	No	–	–	–	5	2

## Discussion

This study found that 55% of Chinese patients with unilateral optic neuritis demonstrated brain lesions and that 48% of the patients had brain lesions that were characteristic of MS. Of these lesions, juxtacortical lesions were the most common. Juxtacortical lesions are as specific for MS as periventricular lesions [12]; however, they were not mentioned in a previous study. The Optic Neuritis Treatment Trial (ONTT) reported that 51% (197/388 patients) of patients had brain white matter abnormalities according to the use of a standardized classification system, including non-characteristic lesions [13]. In a study that used the standardized classification system, a lower percentage (40%) of brain MRI abnormalities was reported in Singapore [14]. The grading system documented only periventricular lesions and brainstem lesions. Data from other Asian regions showed a much lower prevalence of brain MRI abnormalities compared with the figures from the ONTT. In Chinese, Japanese and Taiwanese patients with optic neuritis, periventricular lesions were identified in 8%, 14% and 35% of the patients, respectively [15–17]. These three investigations did not mention juxtacortical lesions or infratentorial lesions, and they applied only MRI T2WI sequences. T2 FLAIR sequences were not available in these studies; therefore, the clinical relevance of juxtacortical lesions might have been underestimated [18]. In our study, a neurologist and a radiologist read the scans using a DICOM viewer program; this method improved the identification of MRI lesions that were characteristic of MS.

In comparison with the high percentage (55%) of patients with brain lesions on the initial scan, the low percentage of patients with Gd-enhanced lesions (3%) or new T2 lesions (10%) on the second scan implies low lesion activity. In contrast to these results, relatively high lesion activity [19], including contrast-enhanced (26%) and new T2 lesions (36%), was observed in a Caucasian population following the identification of a high percentage of patients with brain lesions (78%) on the first scan. During a 5-year follow-up period, more patients (40%) developed CDMS in that study than in ours (18%). Similarly, Rovira et al reported a high level of lesion activity with contrast enhancement (37%) during the first 3 months following onset [20]. In another study, optic neuritis was suggested to be associated with abnormalities on MRI and to be caused by MS [21], especially given the low percentage of Japanese optic neuritis patients (14%) displaying MRI abnormalities [16]. In contrast to that hypothesis, the results of our current study suggest that low lesion activity may be associated with a lower likelihood of conversion to MS.

We found no significant difference in the conversion rate to CDMS between patients with and without brain lesions that were characteristic of MS on the initial scan (21% and 14%, respectively). However, the ONTT [1] reported that brain MRI was a strong predictor of CDMS, with a 5-year risk of CDMS of 16% in 202 patients with no MRI lesions and of 46% in 149 patients with MRI lesions. These CDMS patients exhibited less severe disability during follow-up, although they presented with more severe vision loss than those in the Caucasian population. They had a similar prognosis to the patients observed in previous studies, in whom the MS course was more benign when the initial event was optic neuritis than when the initial event was a brainstem or spinal cord syndrome [22, 23]. Whether high lesion activity on MRI indicates an increased risk of developing CDMS is unresolved. Investigations that include more Chinese patients are warranted.

Chinese patients with unilateral optic neuritis appeared to be more likely to convert to CDMS with relapsing optic neuritis or optic-spinal syndrome. However, in Caucasian patients, the clinical manifestations of CDMS are quite variable due to its progressive nature and the widespread distribution of MS lesions in the gray and white matter of the human central nervous system. The results from one patient with a longitudinal spinal lesion that extended across fewer than 3 spinal cord segments are suggestive of optic-spinal MS. However, the optic-spinal MS patients often present with transverse myelitis [24]. Recently, it has been suggested that optic-spinal MS is historically important but that this term has been superseded [25] because of the use of novel techniques, including MRI criteria for determining the length of spinal cord lesions and anti-AQP4 Ab measurement. Patients with neuromyelitis optica who had a score of 3 or lower on the Expanded Disability Status Scale were considered to suffer from a possibly benign form of neuromyelitis optica, although that conclusion has not been definitively confirmed because a disabling attack can occur after a long follow-up period [26]. In our study, 4 patients developed partial myelitis and anti-AQP4 Ab seronegativity, and these findings are inconsistent with the presence of a benign form of neuromyelitis optica.

This study has some limitations. First, our study included a small sample size. However, the significantly low lesion activity on brain MRI prevented us from enrolling more patients. A previous study showed that in most cases, conversion to CDMS occurred within 5 years of onset of optic neuritis [2]. To reduce bias, we followed these patients for a median of 5 years. No patient received any disease-modifying drug. Therefore, effects of drugs on the disease course were avoided. We also excluded patients with atypical disease courses by applying exclusion criteria. The MRI protocol (slice thickness of 5 or 6 mm) in our study was not optimal for MS lesions [27], and the MRI readers were not double-blinded. We did not routinely perform spinal MRI, although 8 patients displayed normal cervical cord MRI findings upon the initial attack of optic neuritis. That result might have led to bias in our observations of disease activity in the central nervous system.

## Conclusions

Brain lesions characteristic of MS are common in Chinese patients with unilateral optic neuritis; however, these patients display low lesion activity. The predictive value of brain lesion activity for CDMS should be investigated in additional patients.

## Supporting Information

S1 Fig Optic nerve and brain lesions. A coronal STIR sequence showed a hypertensive optic nerve lesion in the left eye (A). Sagittal T2 FLAIR sequences showed a juxtacortical lesion (B) and a periventricular lesion (C). Axial T2WI showed a brainstem lesion (D). An initial MRI scan displaying multiple lesions (E) and a follow-up scan showing a new hypertensive lesion in addition to the old lesions (F).
(TIF)

S2 Fig Cervical spinal cord lesion. Sagittal T2 FRFSE sequences showed a cervical cord lesion (A) that was longitudinally extended across fewer than 3 spinal cord segments. Axial T2 FRFSE sequences (B) showed a lesion located on the left side of the spinal cord.
(TIF)
