# Supplementary figures and images for: Lesion Activity on Brain MRI in a Chinese Population with Unilateral Optic Neuritis
Source: PLoS One. 2015 Oct 20;10(10):e0141005. doi: 10.1371/journal.pone.0141005 (PMC4616383; doi:10.1371/journal.pone.0141005)

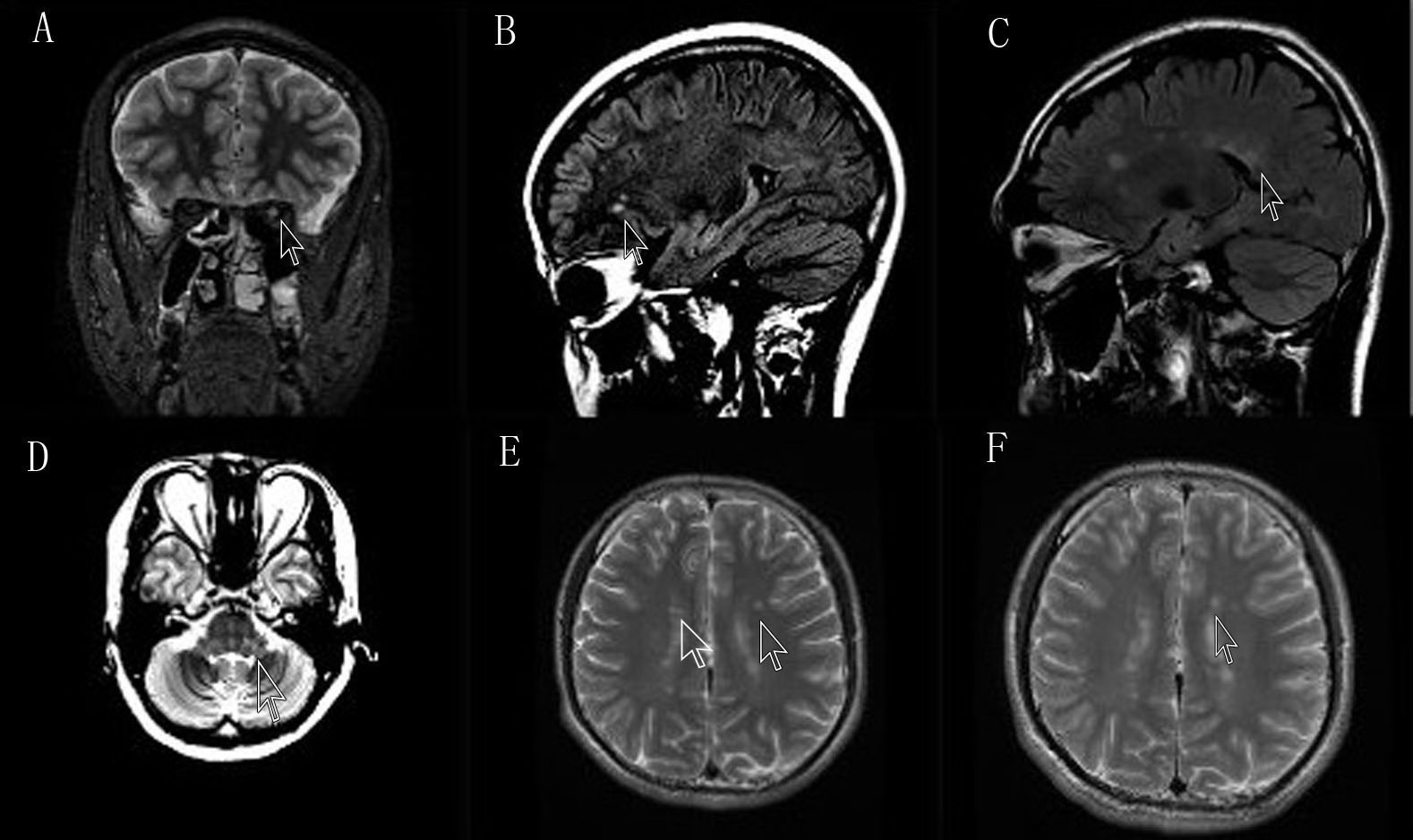

Supplement: S1 Fig — A coronal STIR sequence showed a hypertensive optic nerve lesion in the left eye (A). Sagittal T2 FLAIR sequences showed a juxtacortical lesion (B) and a periventricular lesion (C). Axial T2WI showed a brainstem lesion (D). An initial MRI scan displaying multiple lesions (E) and a follow-up scan showing a new hypertensive lesion in addition to the old lesions (F). (TIF) [file pone.0141005.s001.tif]

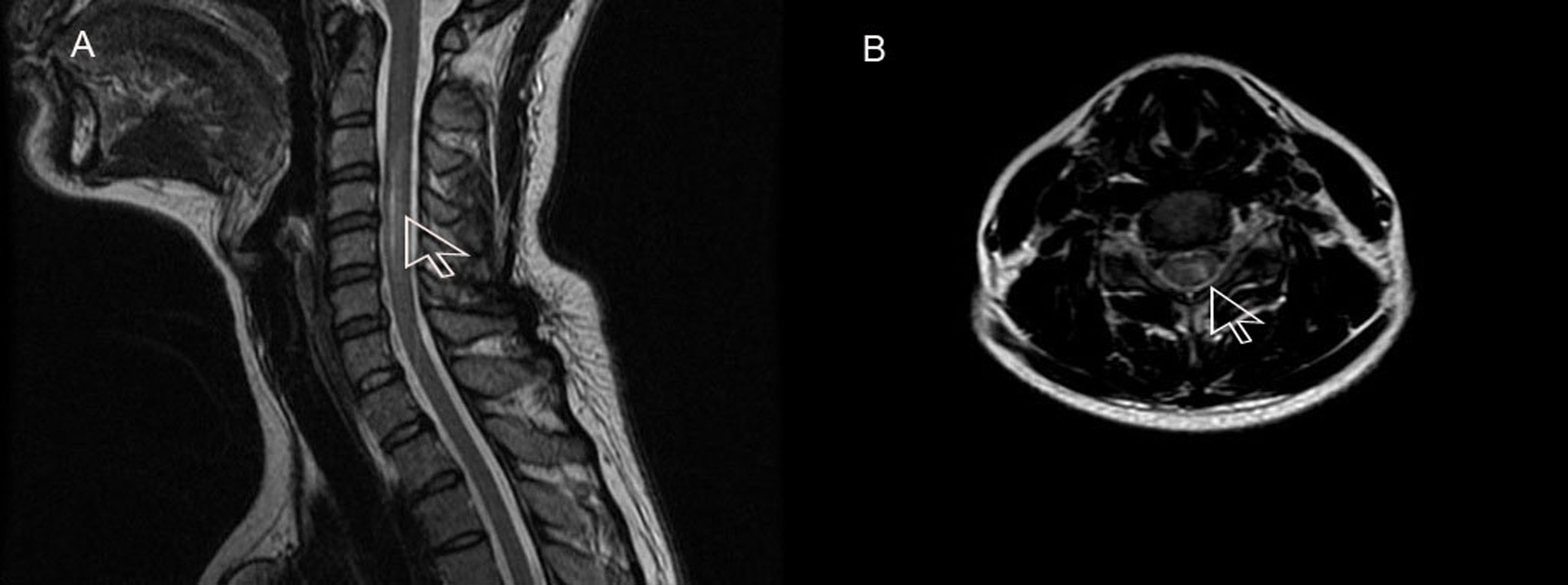

Supplement: S2 Fig — Sagittal T2 FRFSE sequences showed a cervical cord lesion (A) that was longitudinally extended across fewer than 3 spinal cord segments. Axial T2 FRFSE sequences (B) showed a lesion located on the left side of the spinal cord. (TIF) [file pone.0141005.s002.tif]
